# Identifying the Crucial Biomarker of MASH-Related HCC

**DOI:** 10.2174/0113862073287250240424073108

**Published:** 2024-05-09

**Authors:** Haiyang Zhou, Yinjie Zhang, Yuhang Shen, Shuai Chen, Zhen Yang, Zhihuai Wang, Xihu Qin, Chunfu Zhu

**Affiliations:** 1 Department of General Surgery, The Affiliated Changzhou No. 2 People’s Hospital of Nanjing Medical University, Changzhou, 213000, China;; 2 Nanjing Medical University, Nanjing, 210000, Jiangsu Province, China;; 3 Dalian Medical University, Dalian, 116000, Liaoning Province, China;; 4 Center of Gastrointestinal Diseases, The Affiliated Changzhou Second People’s Hospital of Nanjing Medical University, Changzhou, 213000, China

**Keywords:** SPP1, hepatocellular carcinoma, metabolic associated steatohepatitis, immune, immune checkpoints

## Abstract

**Objectives:**

This study aimed to explore the key oncogenic factor of metabolic-associated steatohepatitis (MASH) to hepatocellular carcinoma (HCC).

**Methods:**

We utilized four differential GEO datasets (GSE164760, GSE139602, GSE197112, and GSE49541) to identify the key oncogenic factor for MASH-related HCC. The differential genes were analyzed using the GEO2R algorithm online. The GEPIA online website was used to explore the expression of selected four genes (SPP1, GNMT, CLDN11, and THBS2). The genetic alterations in genes were estimated by the cBioPortal website. The Kaplan-Meier Plotter online database was applied to explore the prognostic value of SPP1. Univariate and multivariate Cox analyses were carried out to further confirm the prognostic value of SPP1. The GO and KEGG enrichment analysis exported associated pathways with SPP1 expression. The positively or negatively related immune cells and immune checkpoint expressions were identified through Pearson correlation analysis. The lipogenesis-associated proteins were detected using western blotting and fluorescence. The high-fat diet (HFD) mouse model was constructed, and liver samples were collected.

**Results:**

SPP1, GNMT, CLDN11, and THBS2 were determined in the transformation process of MASH to liver fibrosis. SPP1 and GNMT were upregulated in the HCC tumor tissue. SPP1, in particular, had the potential to be the prognostic factor through Cox analysis. Remarkably, SPP1 was highly expressed in HCC compared to normal tissues in three independent datasets (GSE121248, GSE14520, and GSE45267). SPP1 is mainly involved in the amplification and deep deletion mutations. SPP1 was found to be strongly correlated with ANXA2 expression, and ANXA2 was also highly expressed in HCC with significant prognostic performance. Moreover, SPP1 was found to participate in the carcinogenic mechanism and correlate with immune cells and immune checkpoint expression. SPP1 knockdown suppressed the SREBP1 and FASN expressions and increased the SIRT1 expression *in vitro*. Moreover, the HFD model validated the upregulation of SPP1 in the fatty liver *in vivo*.

**Conclusion:**

SPP1 may be the key oncogenic factor for the transformation of MASH to HCC, and it could be a potential immunotherapeutic target in HCC.

## 1 INTRODUCTION

Metabolic dysfunction associated steatotic liver disease (MASLD) has been a threatening common disease caused by diabetes and obesity [1]. Especially in developed countries, it has been the main public health problem. Recent studies have reported that MASLD originates from hepatic steatosis and progressively develops into liver cirrhosis [2]. With the decreasing incidence of HBV-related hepatocellular carcinoma (HCC) and HCV-related HCC, MASH is becoming a major cause of HCC [3]. Once liver cancer is advanced, chemotherapy, immunotherapy, and surgery are required, which seriously affects the prognosis of the disease [4, 5]. Therefore, screening the detective biomarker for MASLD-related HCC is necessary for early identification.

Rapid progress in the sequencing technique and genome research has been witnessed for MASLD. MASLD is a common disease that is caused by many factors [6]. It originates from liver cell steatosis and develops into cirrhosis, which has a high risk of changing to (HCC) [7]. It has been reported that increasing morbidity of MASLD-related HCC appears because of obesity [8]. Accumulating evidence attempts to focus on the development and prevention of MASLD-related HCC [9]. Li *et al*. found that mTORC1 is mediated through SREBP1 transcription to influence the pathophysiological process of MASLD-related HCC [10].

In this study, we utilized four public GEO datasets to obtain four genes (SPP1, GNMT, CLDN11, and THBS2) affecting the transformation of MASH to hepatic fibrosis. Clinical information and transcriptome sequencing data of HCC were employed to conduct the Cox regression analysis. SPP1 was found to be the only gene that has prognostic value both in univariate and multivariate analyses. Kaplan-Meier curves indicated that high SPP1 expression is associated with a worse prognosis in HCC patients. Other independent datasets (GSE121248, GSE14520, and GSE45267) further validated the high SPP1 expression in HCC tissue compared to normal tissue. The amplification alteration in the genome was detected by the CbioPortal online website, and ANXA2 may be the downstream gene that could lead to the carcinogenesis of HCC. The expression of lipogenesis-associated proteins all changed after performing the knockdown of SPP1 in HepG2 cells, and the upregulation of SPP1 could be detected in the HFD mouse model.

## 2 MATERIALS AND METHODS

### 2.1 Data Acquisition

The transcriptome data and corresponding clinical information were acquired from the GEO online database (https://www.ncbi.nlm.nih.gov/geo/). The data was downloaded in FPKM format and normalized finally.

### 2.2 Cell Culture

The hepG2 cell line was purchased from Shanghai Fuxiang Biotechnology Co., Ltd. The cells were cultured with DMEM medium, including 10% fetal bovine serum (Gibco, Lofer, Austria) and penicillin-streptomycin solution. All the cells were cultured with 5% CO_2_ in air at 37 °C.

### 2.3 Cell Induction Differentiation

HepG2 cells were cultured in the conventional medium with 500 um of FFA (oleic and palmitic acid, 2: 1) at 37°C and 5% CO_2_ for 24 h to induce their differentiation and stimulate the cells to complete lipid accumulation.

### 2.4 Cell Transfection

HepG2 cells were fully prepared and seeded in a six-well plate, and then siRNA and NC control, which was purchased from RiboBio company, were transfected into the cells with a transfection reagent. All the operations were carried out in accordance with the manufacturer’s instructions. The cells were collected, and RNA was extracted after 48 hours of transfection.

### 2.5 Animals and Treatments

Mice used in the experiments were adult male C57BL/6 mice aged 4−5 weeks. The C57BL/6 mice utilized in this experiment were procured from Changzhou Cavens Experimental Animal Co., LTD. All procedures were approved by Animal Care and complied with legal provisions and national guidelines for the care and maintenance of laboratory animals. Animals were housed in a specific pathogen-free environment with a stationary temperature at 22 ±1°C and a 12 h dark/light cycle. C57BL/6 mice were equally divided into two groups and had free access to food and water after a week of adaptation. The control group was fed with a chow diet (CH), and the model group was fed with a high-fat diet (HF) for 20 weeks. At the end of 20 weeks, mice were sacrificed with pentobarbital sodium, and their livers were rapidly taken out from the abdominal cavity. Some specimens were used immediately or frozen at −80°C for the detection of molecular biological changes or transcriptome sequencing. Meanwhile, other parts of mouse liver tissues were fixed with 10% formalin for storage. Subsequently, they were embedded in paraffin, sectioned at 4 μm, and stained with Sirius red.

### 2.6 qRT-PCR Analysis

The total RNA was extracted using the total RNA TriPure isolation reagent kit (BioTeke, Beijing, China). RNA was reversely transcribed into cDNA using HiscriptII Q RT SuperMix for qPCR with a gDNA wiper (Vazyme Biotech). The process of quantitative real-time PCR proceeded with AceQ qPCR SYBR Green Master Mix Kit (Vazyme Biotech, Nanjing, China) in the ABI 7500 RT-PCR system.

### 2.7 Western Blotting

Liver tissue was homogenized or the cells were lysed for 5 min on ice with Radio Immunoprecipitation Assay (RIPA) lysate. SDS-PAGE was used to separate the proteins on 8% or 10% gels, and then Polyvinylidene Fluoride (PVDF) membranes (MerckMilipore GER) were used to separate the proteins. Then, the membranes were blocked in QuickBlock™ Western sealing fluid (Beyotime Biotechnology, Shanghai, China) for 2 h. The sample was then incubated overnight at 4 °C. The membranes were cleaned three times for 5 min each time with Western wash buffer. HRP Goat Anti-Rabbit IgG was incubated (ABclonal Technology, Wuhan, China) for 1 h, followed by three washes with Western wash buffer for 5 min each. A chemiluminescence system was used to detect signals, and ImageJ Lab was utilized to analyze the data.

### 2.8 Immunofluorescence Analysis

The expressions of lipogenesis-associated proteins (SREBP1, FASN, and SIRT1) were identified using a fluorescent inverted microscope after incubating with an antibody. The operation process was conducted exactly according to the instructions [11].

### 2.9 Statistical Analysis

All of the data analysis was carried out in the context of SPSS 28.0 and GraphPad Prism software operation. Each experiment was repeated three times. *P* value < 0.05 was considered to be statistically significant.

## 3 RESULTS

### 3.1 The Differently Expressed Genes (DEGs) in the Transformation Process of MASH to Liver Fibrosis

**Fig. (1):**
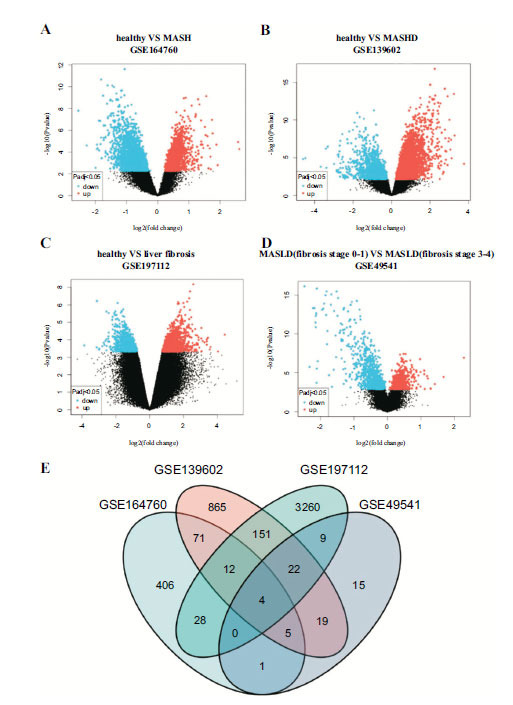
(**A**). The differently expressed genes between MASH samples and healthy control in the GSE164760 dataset. (**B**). The differently expressed genes between liver fibrosis samples and healthy control in the GSE139602 dataset. (**C**). The differently expressed genes between liver fibrosis samples and healthy control in the GSE197112 dataset. (**D**). The differently expressed genes between liver fibrosis samples (stage 0-1) and healthy control (stage 0-1) 3-4) in GSE49541 dataset. (**E**) Intersection of various differential genes in GSE164760, GSE164760,GSE197112,GSE49541. **Abbreviation:** MASH: Metabolic associated steatohepatitis.

The DEGs between healthy people and MASH samples were determined in the GSE164760 dataset (Fig. **1A**). The DEGs between healthy people and liver fibrosis were identified based on GSE139602 and GSE197112 datasets (Fig. **1B**-**C**). The volcano plot was drawn in the GSE49541 dataset by comparing the differential expressions of liver fibrosis of 0-1 stage and liver fibrosis of 3-4 stages (Fig. **1D**). Four genes (SPP1, GNMT, CLDN11, and THBS2) were the intersection genes with the potential to affect the transformation process of MASH to liver fibrosis (Fig. **1E**).

### 3.2 SPP1 Functions as the Key Oncogenic Gene with Prognostic Value

**Fig. (2):**
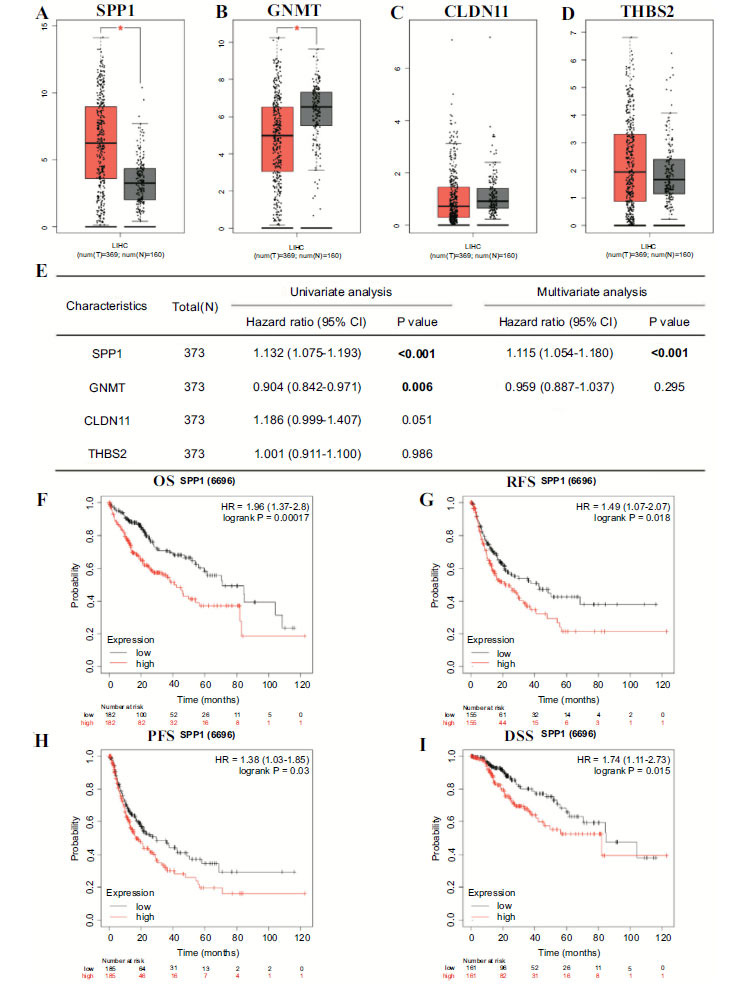
(**A-D**). The differential expressions of SPP1, GNMT, CLDN11, and THBS2 between HCC tissue and normal tissue. (**E**). The univariate and multivariate analysis of SPP1, GNMT, CLDN11, and THBS2 in HCC. (**F**). The differential overall survival (OS) between the high SPP1 expression group and the low SPP1 expression group in HCC. (**G**). The differential recurrence-free survival (RFS) between the high SPP1 expression group and the low SPP1 expression group in HCC. (**H**). The differential progression-free survival (PFS) between the high SPP1 expression group and the low SPP1 expression group in HCC. (**I**). The differential disease-free survival (DSS) between the high SPP1 expression group and the low SPP1 expression group in HCC. **Abbreviation:** OS: overall survival; RFS: recurrence-free survival; PFS: progression-free survival; DSS: disease-free survival.

GEPIA online website was further employed to validate the expression level of the four genes (SPP1, GNMT, CLDN11, and THBS2) in HCC tissue and normal tissue. Compared to the corresponding normal tissue, SPP1 was highly expressed in tumor tissues (Fig. **2A**). Moreover, GNMT was highly expressed in normal tissues (Fig. **2B**). CLDN11 and THBS2 had no significant difference between tumor tissues and normal tissues (Fig. **2C**-**D**). Univariate Cox analysis results showed that SPP1 and GNMT could be the prognostic factor in HCC, and multivariate Cox analysis results suggested that SPP1 is the only gene with the potential independent prognostic value for HCC patients (Fig. **2E**). Kaplan-Meier plotter online website further verified the prognostic significance of SPP1 (Fig. **2F**-**I**), including its impact on the overall survival (OS), recurrence-free survival (RFS), progression-free survival (PFS), and disease-free survival (DSS).

### 3.3 SPP1 Correlated with ANXA2 in the HCC Carcinogenesis Process 

**Fig. (3):**
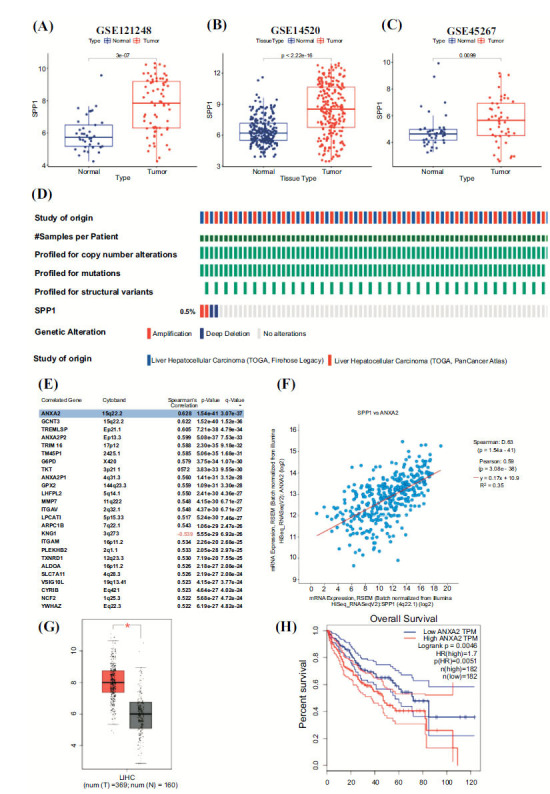
(**A**). The differential expression of SPP1 between HCC tissue and normal tissue in the GSE121248 dataset. (**B**). The differential expression of SPP1 between HCC tissue and normal tissue in the GSE14520 dataset. (**C**). The differential expression of SPP1 between HCC tissue and normal tissue in the GSE45267 dataset. (**D**). The mutation map of SPP1 on the CbioPortal website. (**E**). The top 25 genes correlated with SPP1 expression. (**F**). SPP1 is strongly correlated with ANXA2 expression. (**G**). The differential expression of ANXA2 between HCC tissue and normal tissue. (**H**). The differential OS between the high ANXA2 expression group and the low ANXA2 expression group.

To further ensure the regulation of SPP1 in HCC, three other independent GEO datasets were utilized to explore the expression of SPP1 in HCC tissue (GSE121248, *p*=3E-07; GSE121248, *p*= 2.22E-16; GSE45267, *p*= 0.0099; Fig. **3A**-**C**). The main mutation type of SPP1 was detected by using the CbioPortal online website (Fig. **3D**), and the 0.5% amplification and deletion were detected in the mutation map. To Figure out the potential downstream carcinogenic factor of SPP1 in HCC, the CbioPortal online website found that ANXA2 was the gene that had the most strong correlation with SPP1 (Fig. **3E**-**F**). GEPIA online website indicated that ANXA2 was highly expressed in HCC tissue (Fig. **3G**), and the high ANXA2 expression was correlated with worse survival in HCC (Fig. **3H**).

### 3.4 SPP1 Strongly Correlated with Immune Infiltration

**Fig. (4):**
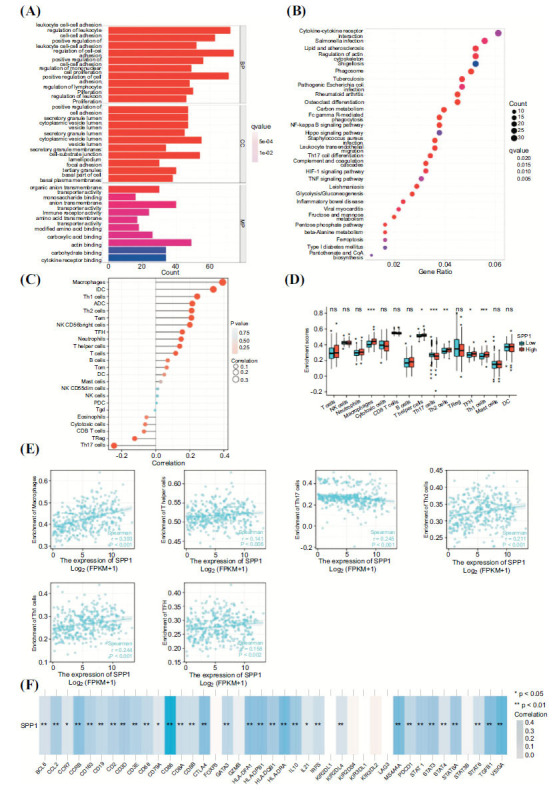
(**A**). The GO enrichment analysis terms of SPP1. (**B**). The KEGG enrichment analysis terms of SPP1. (**C**). The various immune cells related to SPP1. (**D**). The differential infiltration of various immune cells between the high SPP1 expression group and the high SPP1 expression group. (**E**). The expression of SPP1 was correlated with various infiltrating immune cells, including macrophage, T helper cell, Th17 cell, Th2 cell, Tfh, and Th1. (**F**). SPP1 expression was strongly correlated with several immune checkpoints expression.

The GO enrichment analysis indicated that SPP1 was correlated with many carcinogenic and immune-related biological processes (leukocyte cell-cell adhesion, regulation of leukocyte, positive regulation of leukocyte cell-cell adhesion, regulation of lymphocyte proliferation) (Fig. **4A**). KEGG enrichment analysis suggested that various immune-related pathways (NF-κB signaling pathway, Th17 cell differentiation, HIF-1 signaling pathway, and leukocyte transendothelial migration), were related to the SPP1 expression (Fig. **4B**). The Pearson correlation analysis indicated that SPP1 was positively correlated with the infiltration of macrophages, iDC, Th1 cells, and aDc, and it was negatively correlated with Th17 cell and Treg infiltration (Fig. **4C**). Macrophage, T helper cell, Th17 cell, Th2 cell, Tfh, and Th1 cells were highly expressed in the high SPP1 expression group compared to the other group (Fig. **4D**). The scatter diagram shows the strong correlation between the infiltrating level of macrophage, T helper cell, Th17 cell, Th2 cell, Tfh, Th1, and SPP1 expression level in HCC (Fig. **4E**). Moreover, SPP1 expression was strongly correlated with several immune checkpoints expression (BCL6, CCL2, CCR7, CCR8, CD163, CD19, CD2, CD3D, CD3E,CD68, CD79A, CD86, CD8A, CD8B, CTLA-4, GATA3, HLA-DPA1, HLA-DPB1, HLA-DQB1, HLA-DRA, IL10, IL21, IRF5, KIR2DL4, MS4A4A, PDCD1, STAT1, STAT3, STAT4, STAT5A, STAT6, TGFB1 and VSIG4; Fig. **4F**).

### 3.5 The Knockdown of SPP1 Influences the Expression of Lipogenesis-Associated Proteins

**Fig. (5):**
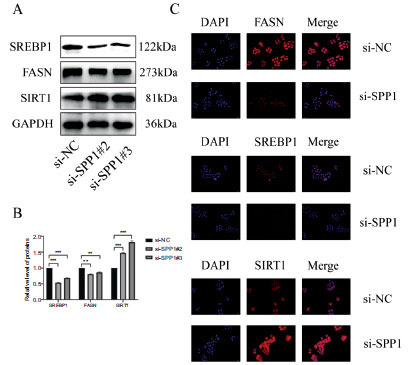
(**A-B**). The Western blot assay showed the protein expression of SREBP1, FASN, and SIRT1 in the si-SPP1 group and the si-NC group. (**C**). The immunofluorescence assay showed protein expression of SREBP1, FASN, and SIRT1 in the si-SPP1 group and the si-NC group.

HepG2 cells were transfected with si-NC or si-SPP1. The result of the western blot assay showed that the protein expressions of SREBP1 and FASN were decreased in the si-SPP1 group compared to the si-NC group, and the protein expression of SIRT1 was increased in the si-SPP1 group compared to the si-NC group (Fig. **5A**-**B**). The immunofluorescence experiment showed that the SREBP1 and FASN expressions were suppressed in the si-SPP1 group, while SIRT1 was upregulated in the si-NC group (Fig. **5C**).

### 3.6 The Transcriptome Sequencing Enriched Lipid Metabolism-Related Pathways

**Fig. (6):**
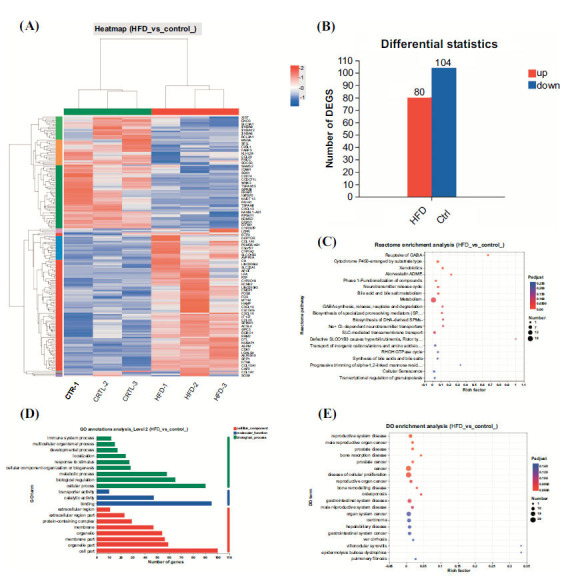
The transcriptomic sequencing of mouse fatty liver samples. (**A**). The heatmap presented the differential expressed genes (DEGs) between the HFD group and the control group. (**B**). The numerical statistics for the DEGs of the control group and HFD group. (**C**). The reactome enrichment analysis of DEGs between HFD and control group. (**D**). The GO annotations analysis of DEGs between HFD and control group. (**E**). The DO enrichment analysis of DEGs between HFD and control group.

The HFD model was constructed, and the samples were used for RNA transcriptome sequencing analysis (Fig. **6A**). The analyzed results showed that 80 genes were up-regulated in the HFD group compared to the control group, and 104 genes were down-regulated in the HFD group (Fig. **6B**). The DEGs were utilized to proceed with the pathways enrichment analysis. The reactome enrichment analysis results indicated that the DEGs mainly enriched in the metabolism (Fig. **6C**). The GO annotations analysis of DEGs between HFD and the control group showed that the top enriched biological process was a cellular process, the mostly enriched molecular function was binding, and the cell part was the highest enriched cellular component (Fig. **6D**). The Disease Ontology (DO) enrichment analysis of DEGs showed that cancer, disease of cellular proliferation, hepatobiliary disease, and liver cirrhosis were the main diseases that DEGs enriched (Fig. **6E**).

### 3.7 Spp1 Expressed More in MASH

**Fig. (7):**
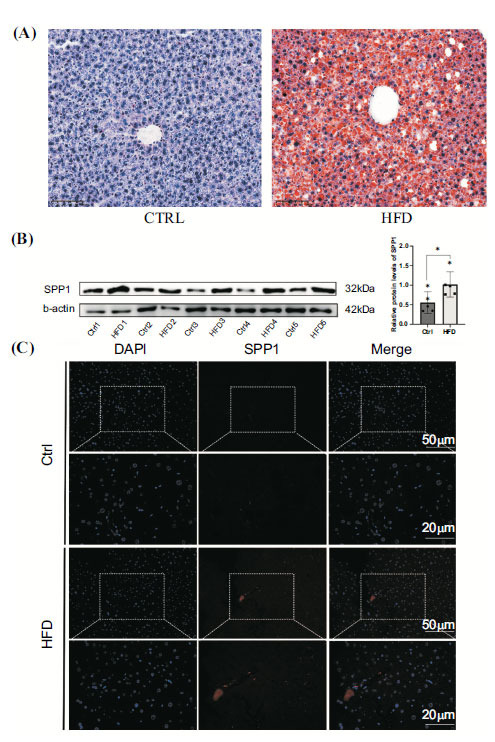
(**A**). The presentation of Oil Red O Staining in the control group and HFD group. (**B**). The Western blotting assay showed the elevated expression of SPP1 in HFD mouse tissue. (**C**). The immunofluorescence assay showed that SPP1 is highly expressed in HFD mouse tissue.

Sirius red staining revealed a higher degree of fatty liver following high-fat diet treatment than the control group (Fig. **7A**). To illustrate the significance of SPP1 in the HFD mouse model, we detected Spp1 expression in mouse MASH hepatic tissues (n=5) and normal hepatic tissues (*n*=5) with WB, which showed a significant increase in Spp1 (p < 0.05) (Fig. **7B**). Similar changes in Spp1 were also verified using immunofluorescence staining (Fig. **7C**).

## 4 DISCUSSION

The morbidity and lethality of MASH-related HCC are increasing yearly [12]. MASH is a type of disease caused by hepatocyte steatosis and inflammation, and it has the potential to lead to cirrhosis even to HCC [13]. The lipotoxicity of hepatocyte steatosis is involved in the process of hepatocarcinogenesis [3]. In this study, we aimed to explore the effective biomarkers involved in hepatocarcinogenesis from the perspective of MASH. We performed various bioinformatics analyses and conducted *in vitro* experiments to validate the carcinogenic role of SPP1 in MASH.

Current research has focused on SPP1 in the pathological process of MASH. Wang *et al*. found that SPP1 could be a key gene associated with MASH-associated HCC, which can also predict the occurrence of HCC [14]. Recently, one research used the bioinformatic and experimental methods to identify the upregulation of SPP1 in MASH patients and mouse models [15]. Tang *et al*. determined the differently expressed genes between healthy control and MASH samples through RNA sequencing, and the MASLD mouse model further verified the overexpression of SPP1 in MASLD [16]. He *et al*. found that SPP1 is one of the upregulated genes in MASH and may contribute to the MASH progression [17]. SPP1 was also reported to be upregulated with the progression of MASLD histology [18]. Accumulating research mentioned the key effect on the tumor transformation, especially the deficiency of SPP1 in mouse model, suggesting that the SPP1 knockdown can inhibit the transformation from MASLD to HCC [19]. In this regard, the carcinogenesis of SPP1 in HCC is also recently highlighted. Liu *et al*. found that SPP1 can induce macrophage polarization in HCC [20]. SPP1 can promote tumor cell proliferation and tumor growth in HCC [21]. Moreover, SPP1 was found to be correlated with metastasis and prognosis of postoperative HCC patients [22]. Zhang *et al*. found that SPP1 mediates the proliferation of LX-2 cells *via* the TGF-β1/Smads pathway in the pathological process of liver fibrosis.

A tumor microenvironment was recently reported to contribute to the detection of tumor biomarkers. Our study found that the expression of SPP1 was correlated with various infiltrating immune cells, including macrophage, T helper cell, Th17 cell, Th2 cell, Tfh, and Th1. Macrophage is a powerful immunoeffector that could promote tumor progression or inhibit tumor development [23]. Pu *et al*. found that extracellular vesicles originating from M2 macrophage can mediate CD8+ T cell exhaustion in HCC, and it directs the new orientation on immunotherapeutic target [24]. Th17 cells are a kind of CD4+ T cell that affects different cancers by mediating IL-17 [25]. Fathi *et al*. found that Th2 cells decreased in cancer patients compared to healthy control, and it may impact the pathological process [26]. The dominance of Th2 cells may mediate the carcinogenesis of HCV-related liver cirrhosis [27]. Our study determined that SPP1 is associated with the immune infiltration of the above-mentioned immune cells in the HCC tumor immune microenvironment. Extant literature further validated that SPP1 plays an important role in the carcinogenic process of HCC.

We identified the key oncogenic factors of MASH-related HCC from four GEO datasets (GSE164760, GSE139602, GSE197112, and GSE49541) using bioinformatics techniques. Furthermore, SPP1 was found to have a better prognostic value for HCC by univariate and multifactorial analyses. In order to verify the specific biological functions involved in the regulation of SPP1, relevant experimental demonstrations were performed at the cellular level. First, the knockdown of SPP1 in HepG2 cells completing lipid accumulation revealed a significant decrease in the protein expression levels of FASN and SREBP1 and an increase in the protein expression level of SIRT1 in the cells, indicating that SPP1 is involved in processes related to hepatocyte lipid accumulation. We further constructed the HFD mouse model and collected the liver sample, and the high Spp1 expression in the liver tissue of the HFD mouse was verified *in vivo*. The transcriptomic sequencing analysis suggested that the DEGs between the HFD sample and control sample were mainly enriched in several metabolism-related pathways and hepatobiliary diseases. Based on these results, it can be concluded that SPP1 plays an important role in regulating leukocyte adhesion and also has a positive effect on the regulation of the immune microenvironment. In liver cancer, SPP1 may be involved in the regulation of immune response through several signaling pathways, such as the NF-κB signaling pathway, Th17 cell differentiation, and HIF-1, thus promoting the progression of the disease. The above experiments suggest that SPP1 plays an important role in all key aspects of the transformation process from MASH to HCC and is a potential marker with multiple biological functions, which has the value of further exploring its involvement in deep-seated regulatory mechanisms. In the future, we hope to develop drugs that target upstream or downstream components of SPP1-related signaling pathways or protein interaction networks. We hope to reveal more about the important role that this protein and its related pathways play in tumor development and bring new breakthroughs to the field of precision medicine.

However, our study still has some limitations. First of all, only validation of *in vitro* cell function experiments was performed in our study. We should enroll more mice to conduct the *in vivo* experiment to verify the specific carcinogenic mechanism of SPP1 in HCC. Secondly, the specific downstream pathways of SPP1 still need to be further explored in detail from MASH to HCC. It will be our future research topic.

## CONCLUSION

Our study found a novel biomarker (SPP1) in the pathogenic process of MASLD-related HCC, and its biological functions correlated with the expression of lipogenesis-associated proteins were identified by detailed exploration.

## Data Availability

The data used in the part of bioinformatics analysis in this manuscript were taken from online databases. All the clinical samples were obtained from the Jiangsu Kebiao Medical Technology Group Co., LTD, with permission from the ethics council of Jiangsu Kebiao Medical Technology Group Co., LTD.

## LIST OF ABBREVIATIONS

HCC: Hepatocellular Carcinoma
HFD: High-Fat Diet
MASH: Metabolic-Associated Steatohepatitis
MASLD: Metabolic Dysfunction Associated Steatotic Liver Disease
